# Factors Associated With Undergraduate Nursing Students' Academic and Clinical Performance: A Mixed-Methods Study

**DOI:** 10.3389/fmed.2022.793591

**Published:** 2022-02-16

**Authors:** Ensieh Fooladi, Md Nazmul Karim, Sheila Vance, Lorraine Walker, Maya Ebrahimi Zanjani, Dragan Ilic, Gabrielle Brand

**Affiliations:** ^1^Faculty of Medicine, Nursing and Health Sciences, Monash School of Nursing and Midwifery (MNM), Monash University, Clayton, VIC, Australia; ^2^Medical Education and Research Quality Unit (MERQ), School of Public Health and Preventive Medicine, Monash University, Clayton, VIC, Australia; ^3^Student Academic Support Unit (SASU), Faculty of Medicine, Nursing and Health Sciences (FMNHS) Education Portfolio, Monash University, Clayton, VIC, Australia; ^4^Monash Centre for Scholarship in Health Education (MCSHE), Monash University, Clayton, VIC, Australia

**Keywords:** nursing education, clinical performance, academic performance, bachelor of nursing students, support structures, educator qualities, interactive curriculum

## Abstract

**Background:**

There is conflicting and limited information regarding factors that influence undergraduate nursing students' academic and clinical performance prior to entry to practice.

**Objective:**

To identify factors influencing the academic and clinical performance of undergraduate nursing students throughout the course.

**Design:**

Mixed methods study utilizing a retrospective cohort and a qualitative study.

**Setting:**

Monash University, Melbourne, Australia.

**Participants:**

Longitudinal existing data of nursing undergraduate students who commenced in 2017 (*n* = 176) and 2018 (*n* = 76), and two focus groups with final year nursing students were analyzed.

**Methods:**

Retrospective students' records were used to determine the students' academic and clinical performance using the weighted average mark (WAM) of the theoretical and clinical components of the curriculum, separately. The WAM considered the year level of each unit and was scored out of 100. Multivariate linear regression was used to determine predictor factors of academic and clinical performance. Variables include entry cohort (with no previous nursing qualification vs. diploma of nursing), admission category (domestic vs. international), campus (metropolitan vs. outer metropolitan), and secondary school (year 12) results. Two focus group discussions were conducted and thematically analyzed.

**Results:**

More than two-thirds of the students were aged 18–20 years and mainly female. Almost 20% of the participants were international students. Students with higher secondary school (year 12) results and studying at the outer metropolitan campus achieved a higher academic performance while international students had significantly lower academic performance compared to domestic students. Students with a previous diploma of enrolled nursing and international students had lower clinical performance. Students identified that a comprehensive orientation, interactive curriculum, formal and informal support structure, and educator qualities influenced their academic and/or clinical performance.

**Conclusions:**

A supportive educational environment with an interactive curriculum may enhance students' academic and clinical performance and readiness for practice. Furthermore, targeted interventions for international students, those with lower secondary school (year 12) results, and those with a former diploma of nursing may be required to increase academic and clinical performance.

## Introduction

With the recently celebrated International Year of the Nurse, it is timely to consider the education of tomorrow's nursing workforce in increasingly difficult and complex environments. To promote excellence in nursing, Darbyshire et al. [(1), p. 2] suggest that faculties need to “develop and sustain a culture in which excellence in scholarship can flourish and deliver responsive, challenging educational programs.” The question of whether universities are providing education programs that prepare students with sufficient levels of academic and clinical capability is of vital importance to the future of the nursing profession. One strategy to address this issue is to identify factors that facilitate nursing students' academic and clinical performance success.

An increasing number of students enrolling in Bachelor of Nursing (BN) courses are older than 21 years of age (2) and come from diverse academic, ethnic, and cultural backgrounds (3). Pitt et al. (4) examined the predictive nature of demographic, academic, and personality/behavioral factors in determining the academic success of nursing students, concluding that information on key factors impacting clinical performance is scarce. While academic outcomes for different entry pathways into a BN program have been studied, other influences, such as student background, have not been explored (5). To date, only one Australian study has examined factors such as grade point average (GPA) (the number signifying the median value of the accrued final course grades) and domestic or international status (6). Findings from that study concluded that international students, and those with a lower GPA, were more likely to demonstrate lower clinical performance (6). Understanding why this occurs is problematic as no qualitative studies have been done to date that explores students' perspectives on the diverse factors that affect academic and clinical performance, including support mechanisms and structures.

The School of Nursing and Midwifery of this higher education institute in Australia offers a 3-year accredited undergraduate nursing course to prepare graduates to be eligible to apply for entry into the nursing profession (7). The BN course employs an integrated active learning curriculum involving scaffolded content over 3 years (8). Assessment in the curriculum was based on best practice assessment development (8). Consistent with the active learning model, assessment activities in the curriculum are learner-centered and designed to reflect a pedagogical approach. Formative and summative assessments are used to encourage learners to act as learning resources for one another reflecting a commitment to collaborative and cooperative learning, reciprocal teaching, and peer assessment. The summative assessment takes an integrated approach that represents a holistic approach to learning. The assessments include quizzes, concept or mind maps, objective structured clinical evaluation (OSCE) skill assessments in a simulation environment, oral presentations, reflective activities, and class participation. Peer-to-peer assessment, abstracts and posters, written assignments, and exams are also included.

The objectives of this study were 1) to examine the impact of demographic characteristics and admission criteria on undergraduate nursing students' academic and clinical performance; 2) to explore final year nursing students' perceptions of factors influencing their academic and clinical performance throughout the course.

## Methods

This mixed methods study utilized quantitative data (nursing student records) to explain the impact of demographic characteristics and admission criteria, and qualitative data (focus group discussions) to explore final year students' perceptions of academic and clinical performance to produce detailed insights (9). Student cohorts were from two Monash Nursing and Midwifery campuses (metropolitan vs. outer metropolitan), in Victoria, Australia who entered *via* two pathways. The first cohort transitioned from secondary school education to commence a 3-year BN in 2017 (*n* = 176). The second cohort, who commenced their 2-year BN in 2018 (*n* = 76), held a diploma of nursing (Enrolled Nurse, EN) and were exempted from the first year. Both groups graduated in December 2019. Of note, all students with a previously enrolled nursing degree were domestic. Students who discontinued other tertiary courses prior to joining the BN course were excluded from the study (*n* = 18) as they might have different knowledge and experiences that might impact their academic and clinical performance.

Demographic and admission information, including admission category (domestic/international), campus location (metropolitan vs. outer metropolitan), age, gender, having a diploma of nursing (yes/no), and secondary school final year 12 (Australian Tertiary Academic Ranking, ATAR) score were retrieved from the Monash Nursing and Midwifery enrolment records. The ATAR score for mature entry students was calculated the same as the other group and was retrieved from administrative students' records.

The weighted average mark (WAM) was used to measure the students' academic, clinical and overall performances (10). The WAM is calculated based on students' actual marks and the year level of each unit and scored out of 100 (10). The WAM is the average mark students achieve across all completed units in a course, including any failed and repeated units. The total WAM, clinical and academic WAM for each year level, and for the whole course, was calculated for each student. The total WAM as a proxy to overall performance was calculated from the total marks obtained in each unit (10). The WAM for clinical performance was calculated using clinical scores (out of 100) obtained in six units with clinical components (10). For example, if a unit has a 20% clinical component, and the student received 18, the clinical mark would be 90% [(18^*^100)/20]. To calculate WAM for academic performance, only marks obtained in theoretical units were used (10). For units with clinical components attached to them, the clinical score was deducted from the total marks. For example, if a students' total mark in the same unit was 75, and their score for the clinical component was 18, the academic mark for this unit would be 75–18 = 57. This 57 score is converted to a percentage using this formula: [(57^*^100)/80] which gives a value of 71.

A clinical portfolio records clinical skills and placement reports. The weighted clinical assessment report utilizes a Bondy scale (11) to allow varying levels of capability to be awarded. The six clinical units have different component percentages attached to placement assessment, including one unit in the first year (20%), three units in the second year (30, 15, and 15%), and three units in the third year (30% each).

A purposeful sample was utilized to recruit participants for our focus group discussion (FGD) by asking BN student representatives at both campuses to advertise the study to year three nursing students *via* the year three-unit Moodle sites (Learning Management System). To recruit participants for our FGD, the study flier was posted on year three-unit Moodle sites (Learning Management System). The students' representatives at both campuses were approached to encourage them to advertise the study to the other year three nursing students they knew. Qualitative data were captured during two, 1-h FGD sessions at metropolitan (*n* = 4) and outer metropolitan (*n* = 9) campuses. All FGDs were conducted by female interviewers, SV and EF, at the metropolitan campus, and by GB and EF at the outer metropolitan campus. None of the interviewers taught into the BN program for these cohorts of students. Students knew that the interviewers are academic members of the Monash Nursing and Midwifery and the Student Academic Support Unit in the Faculty of Medicine, Nursing and Health Sciences. At the start of each focus group, students completed a questionnaire about demographic characteristics and whether they utilized university and/or faculty support services (e.g., Peer Assisted Study Sessions, PASS; Student Academic Support Unit, SASU) (Supplementary Material). The FGD was guided by open, semi-structured questions (Supplementary Material) to explore final year students' perceptions of factors that influenced academic and clinical performance in both positive and negative ways.

### Ethics

The study was approved by the Monash University Human Research Ethics Committee (19337). De-identified data from students' admission and progress records were extracted. The student privacy collection statement allows for the use of the information for research purposes. In addition, we posted a note on one of the Moodle sites of year three nursing students asking them to inform us if they would let us use their demographic, and admission data (e.g., admission qualifications, ATAR score), and all unit scores collected by the University. They were informed that there would be no implications in their personal or academic life for disallowing the use of their data. Informed written consent was obtained for FGD participation which was audio-recorded, and pseudonyms were used for participants.

### Data Analysis

All categorical and continuous variables are described using frequencies (percentages) and means (SDs), respectively. The distribution of the WAM and GPA were assessed, with the assumption of linear regression (12), through generating histogram with normal distribution curve. Correlations between Academic performance and clinical performance WAM across year levels were assessed employing person correlation (13). Pearson correlation coefficient and *p*-values are presented as a correlation matrix. We have generated a histogram for all quantitative variables and a normal distribution curve for checking the skewness. Normality was assessed using Kolmogorov-Smirnov test. All the WAM data were found to be normally distributed. Hence, we used parametric tests. We have adopted a complete case analysis protocol and students with missing data were excluded from the analysis.

The univariate association of gender (male vs. female), student cohort (2017 entry vs. 2018 entry), campus (metropolitan vs. outer metropolitan), and admission category (domestic vs. international) with each of the academic, clinical, and overall performance (WAM) scores was assessed through fitting simple linear regression.

Multivariate linear regression modeling was fitted with each of the academic, clinical, and overall performance (WAM) scores with plausible predictors adjusting for all variables (gender, student cohort, campus, admission category, and year 12 results) simultaneously entered in the model. Analyses were performed using IBM SPSS version 26 (IBM SPSS Statistics for Windows, Version 26.0. Armonk, NY: IBM Corp). All statistical tests were two-sided and the statistical significance was set at the level of 5%.

The FGD data were transcribed verbatim and independently analyzed by three co-authors focusing on ideas and key concepts that stood out from the FGD data. Manual color coding was used to code each FGD to search for meanings and identify thematic threads and patterns (Braun and Clark, 2006). This initial coding and review were then discussed and validated with the remaining authors and distilled into three main themes presented below with student participants' supportive quotations from the FGD transcripts. The trustworthiness of the data was maintained by two authors attending each FGD and debriefing directly after to ensure the accuracy of interpretations of what the participants were saying during the FGD.

## Results

### Quantitative Results

Data from 252 nursing students were used, with 69.8% commencing the course in 2017 and 30.2% in 2018. The mean (SD) age of the students was 21.7 ± 5.4. Student demographics are presented in Table 1.

**Table 1:** Participant's characteristics.

**Characteristics**	
Age, years, median (range)	19.5 (18–56)
**Age, years**, ***n*** **(%)**	
18–20	165 (65.7)
21–25	48 (19.1)
26–30	20 (8.0)
>30	18 (7.2)
**Gender**, ***n*** **(%)**	
Female	221 (87.7)
Male	31 (12.3)
Year 12 results, median (range)	73.8 (20.9–98.2)
**Student cohort**, ***n*** **(%)**	
2017 entry^*^	176 (69.8)
2018 entry	76 (30.2)
**Admission category, n (%)**
Domestic	206 (81.7)
International	46 (18.3)
**Campus**, ***n*** **(%)**	
Metropolitan	136 (54.0)
Outer metropolitan	116 (46.0)
Academic total WAM, mean (SD)	73.0 ± 5.0
Clinical total WAM, mean (SD)	88.2 ± 5.1
Overall WAM, mean (SD)	74.8 ± 4.7

*BN, Bachelor of Nursing; SD, Standard Deviation; WAM, Weighted Average Mark*.

* *2017 entry had no previous nursing qualification*.

The academic and clinical WAMs of year 2 and 3 students were positively correlated with the total academic WAM and overall WAM. No statistically significant correlation was identified between year 1 academic WAMs with yearly WAMs and overall scores. No correlation was observed between year 1 clinical WAMs and overall scores, however, a statistically significant negative correlation was observed between year 1 clinical WAMs with yearly WAMs (Table 2).

**Table 2:** Correlation matrix for yearly and overall WAM of academic and clinical performance.

	**Academic year 2**	**Academic year 3**	**Academic total**	**Overall WAM**
Academic year 1	0.082	0.140^*^	0.079	0.024
Academic year 2		0.736^***^	0.867^***^	0.886^***^
Academic year 3			0.833^***^	0.834^***^
Academic total				0.991^***^
	**Clinical year 2**	**Clinical year 3**	**Clinical total**	**Overall WAM**
Clinical year 1	−0.359^***^	−0.201^**^	0.344^***^	−0.017
Clinical year 2		0.334^***^	0.705^***^	0.328^***^
Clinical year 3			0.473^***^	0.219^**^
Clinical total				0.337^***^

*WAM, Weighted Average Mark*.

* *p < 0.05*.

** *p < 0.01*.

*** *p < 0.001*.

Academic (*p* = 0.646), clinical (*p* = 0.879), and overall (*p* = 0.610) WAM were similar between male and female students. Clinical WAM was found to be significantly higher in the 2017 entry in comparison to the 2018 entry (*p* < 0.001). Academic WAM (*p* = 0.028), clinical WAM (*p* = 0.02), and overall WAM (*p* = 0.001) were significantly higher in domestic students in comparison to the international students (Table 3).

**Table 3:** Univariate association of academic, clinical and overall performances WAM score using simple linear regression.

		**Academic WAM**		**Clinical WAM**		**Overall WAM**	
	***n***	**Mean (SD)**	***P*-value**	**Mean (SD)**	***P*-value**	**Mean (SD)**	***P*-value**
**Gender**							
Male	30	74.4 (5.2)	0.646	88.1 (5.2)	0.879	74.4 (5.1)	0.610
Female	207	74.9 (4.9)		88.2 (5.1)		74.9 (4.6)	
**Student cohort**							
2017 entry^π^	161	74.5 (4.9)	0.157	89.1 (5.1)	<0.001^***^	74.5 (4.5)	0.128
2018 entry	76	75.5 (5.0)		86.2 (4.6)		75.5 (5.0)	
**Campus**							
Metropolitan	126	74.4 (4.6)	0.210	87.8 (5.3)	0.198	74.1 (4.8)	0.008^**^
Outer metropolitan	111	75.3 (5.4)		88.6 (4.9)		75.6 (4.4)	
**Admission category**							
Domestic	195	75.2 (5.1)	0.028^*^	88.6 (5.1)	0.020^*^	75.3 (4.6)	0.001^**^
International	42	73.3 (4.0)		86.6 (5.1)		72.8 (4.5)	

*BN, Bachelor of Nursing; SD, Standard Deviation; WAM, Weighted Average Mark*.

* *p < 0.05*.

** *p < 0.01*.

*** *p < 0.001*.

π *2017 entry had no previous nursing qualification*.

No significant difference in academic (*p* = 0.157) and overall (*p* = 0.128) WAM was identified between the students of 2017 and 2018 entries. However, clinical WAM was significantly higher in 2017 entry students (*p* < 0.001). The academic (*p* = 0.028), clinical (*p* = 0.020), and overall (*p* = 0.001) WAM appeared significantly higher in domestic students when compared to international students. Between the two campuses, students at the outer metropolitan campus (*p* = 0.001) scored a slightly higher WAM than those at the metropolitan campus.

Three multivariate linear regression models assessed the predictors of students' performance (Table 4). Adjusting for all other factors entered in the model, higher year 12 results (β = 0.2, 95% CI.1 to.2); *p* < 0.01) and outer Metropolitan campus (β = 2.0, 95% CI.5 to 3.4; *p* = 0.007) predicted higher academic performance. Conversely, international student status predicted lower academic performance compared to domestic students (β = −2.5, 95% CI −4.8 to −0.2; *p* = 0.034).

**Table 4:** Predictors of academic, clinical and overall performances assessed using multivariable linear regression models.

**Variables**	***n***	**β**	**95% CI**	***p*-value**
**ACADEMIC PERFORMANCE (ACADEMIC WAM AS AN OUTCOME)**				
**Student cohort**				
2017 entry^*^	161	Reference	-	
2018 entry	76	1.8	−0.4 to 3.9	0.103
**Campus**				
Metropolitan	126	Reference	-	
Outer metropolitan	111	2.0	0.5 to 3.4	0.007
**Admission category**				
Domestic	195	Reference	-	
International	42	−2.5	−4.8 to −0.2	0.034
Year 12 results	202	0.2	0.1 to 0.2	<0.001
**CLINICAL PERFORMANCE (CLINICAL WAM AS AN OUTCOME)**				
**Student cohort**				
2017 entry	168	Reference	-	
2018 entry	76	−2.7	−5.0 to −0.4	0.024
**Campus**				
Metropolitan	132	Reference	-	
Outer metropolitan	112	1.0	−0.5 to 2.5	0.203
**Admission category**				
Domestic	198	Reference	-	
International	46	−3.6	−5.9 to −1.3	0.003
Year 12 results	202	0.1	−0.0 to 0.1	0.160
**Total PERFORMANCE (OVERALL WAM AS AN OUTCOME)**				
**Student cohort**				
2017 entry	175	Reference	-	
2018 entry	76	2.4	0.5 to 4.3	0.014
**Campus**				
Metropolitan	136	Reference	-	
Outer metropolitan	115	2.1	0.8 to 3.4	0.002
**Admission category**				
Domestic	175	Reference	-	
International	76	−3.2	−5.2 to −1.2	0.002
Year 12 results	202	0.1	0.1 to 0.2	<0.001

* *2017 entry had no previous nursing qualification*.

For clinical performance, the entry in 2018 (β = −2.7, 95% CI −5.0 to −0.4; *p* = 0.024) and international student status (β = −3.6, 95% CI −5.9 to −1.3; *p* = 0.003) predicted lower clinical performance.

For overall performance, higher year 12 grades (β = 0.1, 95% CI.1 to.2; *p* < 0.001), entry in 2018 (β = −2.4, 95% CI.5 to 4.3; *p* = 0.014), and studying at the outer metropolitan campus (β = 2.1, 95% CI 0.8 to 3.4; *p* = 0.002) predicted higher overall performance. International students demonstrated lowered overall performance compared to domestic students (β = −3.2, 95% CI −5.2 to −1.2; *p* = 0.002).

### Characteristics of the FGDs Participants

The majority of FGD participants were aged between 20and 25 years; 10 of the 13 students were female, and three were international students. Nine students completed the 3-year BN and four the 2-year EN entry-level pathway.

### Qualitative Themes

The three major themes that influenced the nursing students' academic and clinical performance were comprehensive orientation, formal and informal support structures, and educator qualities.

#### Comprehensive Orientation

A comprehensive orientation to the university and clinical life was identified as a positive influence on subsequent performance, especially for international students and enrolled nurses. Students highlighted aspects of academic orientation such as navigating the learning management systems (LMS), library tours, referencing handbook, and peer-assisted study sessions (PASS):

“*It's just the big step up to university ….it's like just to get your bearings and then to practice academic writing and then the Moodle* [LMS] *session and all that sort of stuff just to get your head into it.”* (outer Metropolitan FGD (P-FGD).

This introduction to university life fostered students' connections with their peer group and academic staff. This was particularly important for international students acclimatizing to the Australian culture:

“*Back in the first year I've been to SASU [the Faculty's Student Academic Support Unit] as well. So, it really helped me doing assignment, explain the assignment instructions… So that's really helpful And then I work with English Connect [the University's general English language support service]. So, it pretty much helped me to adapt to Australia, and as well as gain confidence in speaking English Teaching like how to engage with domestic students as well. So that really helped me to make more local friends.”* (Metropolitan FGD (C-FGD).

In the clinical environment, overwhelmingly, the most positive influences were feeling welcomed on clinical placement and having clearly stated guidelines about their scope of practice:

“*… the educator discusses about our scope of practice in the very first day at the orientation, which really benefits. Then we don't have to being a bit scared when I want to take bloods, then I have to page the educator, to double check whether I'm able to doing that or not…”* (C-FGD).

In addition to a comprehensive orientation, insight into clinical performance was enhanced when the university or the health service prioritized formal or informal debriefs following clinical placement too:

“*Designate a time when you finish your practice, come back to have a debrief. So, you hear that everyone else is going through the same thing.”* (P-FGD).

#### The Interactive Curriculum

The interactive curriculum included simulated clinical learning environment (CLE), workshops; peer-assisted, case-based, and small group learning; mentoring and networking, which fostered students' academic performance. Students enjoyed interprofessional education with other health professionals as a mechanism to address any stereotypes and misconceptions, and build communication skills and professional relationships that prompted self-reflection as future professionals:

“*… were quite insightful in terms of providing an idea of what the different roles of health professionals have within the health care system itself. And that also got me to think of how I would be approaching different occupations… how I will be talking in approaching different professionals while studying or working.”* (C-FGD).

Students found the course materials logically organized, with systems-based foundational topics in the first year, and cumulative knowledge and skills refinement in subsequent years increasing in complexity:

“*You will still be applying the theory that you learned in first year, you might be in second year learning a bit more complex nursing care and diseases and conditions.”* (C-FGD).

Students valued a practice-based approach to learning in the context of “real world” scenarios (e.g., case studies), and the opportunity for interaction with others, such as group work.

#### Formal and Informal Support Structures

The course integrates various informal and formal support structures, including PASS mentoring:

“*The content was quite overwhelming for people. They* [PASS tutors] *just really broke it down. You know, just simplified everything for us”* (P-FGD).

The formal peer learning program with final year students supports first-year students in their practical lab sessions:

“*I was asking the questions with them…. So, it would give me like an insight of what I expected going into the placement, and …my nursing journey will be going on.”* (C-FGD).

The clinical support offered to international students by SASU, including simulated role-play, was important in preparing them for clinical placements:

“*So that really helps me to build up the foundation and build the basis of how placement will work and how* [on] *placement I should communicate.”* (C-FGD)

Despite the formal supports, the EN cohort felt that the informal collegial (peer) supports were the most significant factor affecting their academic and clinical performance:

“*Every single person in this room I've contacted at one point or another. Yeah, I wouldn't have finished this course without a good group of friends*.” (P-FGD).

#### Educator Qualities

Students reported that supportive nurse educators helped them learn and *get the best marks* (P-FGD) across both the academic and clinical settings. Educator qualities included being approachable, knowledgeable, fun, setting high academic expectations, providing clinically relevant examples, and being available.

“*We can organize a meet up with one of the educators to actually discuss about that assignment. And that helps to further improve the next assignment that we're going to do. Students would ask questions frequently and would be met with quite quick replies from tutors and lecturers”* (C-FGD).

One student highlighted the impact of having positive role models:

“*a particular lecturer who was just phenomenal …the way that she taught was just so clear and concise … she really pushed me to want to learn.”* (P-FGD).

In contrast, students recounted how unclear assessment tasks, feedback time, and/or inconsistent marking deflated their confidence.

“*I feel like assignment feedback can tend to be quite vague and general, so maybe something like a comment of ‘you were too brief here', but they're not really telling you where”* (C-FGD).

Enhanced clinical performance and learning were dependent on the clinical educators as one student identified that support varied considerably between clinical placement sites:

“*The support of the educators and the senior nurses on the ward greatly impacted how I did on all my placements. There were some placements where I felt less supported … and then I'd go to another placement where full support was given to students, regular check-ups and positive educator attitude toward students… And I think that one was the placement that I got the best out of all ….”* (C-FGD).

For some students their clinical placement resulted in negative learning experiences due to unsupportive behaviors:

“*But there are …nurses who are in there to get their pay and get out. Yeah, so nasty sometimes…I was the only student who didn't end up in the toilets in tears because they've been ridiculed or bullied.”* (P-FGD).

## Discussion

Findings from our mixed methods study highlighted that nursing students with higher secondary school results, a prior EN diploma, or students based on the outer Metropolitan campus achieved a higher total academic performance. Nursing students with a prior EN qualification, or enrolled as international students, had lower academic performances. Our findings support recent studies that have concluded that no single criterion is predictive of academic success in undergraduate nursing programs (14, 15).

Universities traditionally select students based on their year 12 secondary school performance. Our findings support those from studies in Pakistan (16), New Zealand (17), and the USA (18) of a positive predictive validity of secondary school performance with students' academic performance. Conversely, an Australian study (19) found no correlation between secondary school scores and first-year results, although that particular study did not focus on the final course results.

International students in our study scored lower in academic performance when compared to domestic students. While acknowledging the multifaceted nature of academic outcomes, possible explanations for this discrepancy include a lack of familiarity with the Australian education system, English language acculturation (20–22), alongside non-academic, personal, and other contextual factors (23–25).

Similarly, a variety of factors may impact international student performance in the clinical context including knowledge of the Australian health care system, role confusion, and mismatch of expectations between students and clinical staff (25, 26). Cultural issues may also influence performance including differences in cultural values, misunderstanding between students and educators (27–29), and level of assertiveness (30, 31). The use of idiom and slang, the rapid speech and accent of Australian speakers (27, 29, 32), and familiarity with medical terminology, jargon, and language use within specific contexts, such as during a nursing handover (21, 33–35), have also been found to negatively impact on international students' clinical performance.

Clinical supervisors who educate students during clinical placement simultaneously have to provide patient care and constructive learning opportunities which can be impacted by a limited provision of time, reluctance to assign particular tasks, and/or the use of inexperienced preceptors (25). The cultural differences and language backgrounds of the clinical educators or nursing students can also add to this cognitive load, influencing perceptions of challenge and time constraints (27) and may explain why international students scored lower in clinical performance indicators. According to (36), the preceptor (educator) role in the clinical setting also does not consider socio-cultural practice and social learning perspectives, therefore educators' lack of cultural awareness may result in negative assessments of international student behaviors (30, 31). In addition, a term is known as the “halo effect” may lead examiners to focus on first impressions due to differences in accent or minor surface errors in expression, with unsubstantiated extrapolation to a lack of English language proficiency or a lack of knowledge or skills (29).

International students in FGD identified that clear guidelines, expectations, and engagement with accessible, responsive, and empathic educators are central to their academic and clinical performance. Scaffolding and capacity-building aspects of the curriculum, including formal language and targeted academic skills, support, and peer mentoring, were key factors to both their academic and clinical performance success.

Interestingly, this study also found that while ENs performed well academically, they performed less well on clinical placement. This is consistent with recent research that suggests ENs are stressed because they have previous healthcare experience “but then lost this status when becoming a student, resulting in a loss of self-esteem” (37); p. 399 and/or a lack of development of critical thinking, essential for tertiary-level study (37). The ENs transitioning to BN study may also “grapple with their dual identity, have difficulty reconciling their academic and clinical competence, and struggle to assimilate to the academic learning environment” (38); p. 1919. Further research is needed, especially relating to clinical supervisors' perceptions and expectations of ENs compared to BN students whilst on clinical placement. Exploring ENs' transition into BN courses and how it impacts their clinical placement performance also warrants further exploration.

### Limitations and Recommendations for Further Research

The participant selection for the two FGD was voluntary; therefore, a limitation of this study is selection bias (39) as the student participants may have had pre-existing positive or negative perceptions of factors that influenced their academic and clinical performance throughout the course. Moreover, quantitative data from one institute was used which might limit the generalizability of the findings. In addition, the sample size could have been larger; however, we have checked assumptions for correlation and regression prior to the analysis, hence the impact of low sample size is less likely. There are also multiple factors not examined in this study that may affect clinical performance (24, 40), including the students' learning preferences (41), personal qualities, age, and outside employment (4). In addition, the students' personal characteristics (e.g., gender, ethnicity) and qualities of academic and clinical examiners themselves (experience in assessing, site effects) can have an impact on clinical and academic performance (6, 42). Clinical performance measured by grades cannot be always translated to clinical competency. Hence, future longitudinal research is needed to assess the relationship between clinical grades and clinical competency.

The quantitative component of this study is based on demographic data, including the enrolment status category (international or domestic), which has inherent limitations (e.g., some students enrolled as domestic students were born overseas). The international/domestic dichotomy could lead to assumptions about cohort performance that may not be supported. There may also be differences in academic outcomes with international students less likely to withdraw and have a higher GPA than domestic students (43).

Despite the limitations, this study provides valuable insight into the educational needs of our diverse nursing student cohorts. Both international students and ENs are assets to help fill the future nursing workforce shortfall, especially in providing quality patient care outcomes and potentially reducing health discrepancies (28, 40) in the Australian population. Future research should include participants' cultural background, years of study in English, length of residence in Australia, and prior work experience in nursing overseas. Future research could also investigate the educators' experience in assessing students' academic and clinical performance and site-specific factors to ascertain why students in the same course at different campuses scored differently. It would also be interesting to see if other health professions courses have found similar factors influencing students' academic and clinical performance.

## Conclusion

With the national shortage of nurses imminent, higher education institutions need to create targeted education to support diverse undergraduate nursing cohorts. This is particularly important to ensure enrolled nurses' clinical performance success and international nursing students' academic and clinical performance success. Enhancing nursing courses through a comprehensive orientation, embedding formal and informal support structures, and providing quality academic and clinical educators will ensure all BN students can progressively develop the knowledge, skills, and clinical practice required to sustain the Australian nursing workforce.

## Data Availability Statement

The raw data supporting the conclusions of this article will be made available by the authors, without undue reservation.

## Ethics Statement

The studies involving human participants were reviewed and approved by Monash University Human Research Ethics Committee (19337). The patients/participants provided their written informed consent to participate in this study.

## Author Contributions

All authors listed have made a substantial, direct, and intellectual contribution to the work and approved it for publication.

## Funding

The project was funded in 2019 by Monash University Faculty of Medicine, Nursing and Health Sciences Learning and Teaching Research Grant.

## Conflict of Interest

The authors declare that the research was conducted in the absence of any commercial or financial relationships that could be construed as a potential conflict of interest.

## Publisher's Note

All claims expressed in this article are solely those of the authors and do not necessarily represent those of their affiliated organizations, or those of the publisher, the editors and the reviewers. Any product that may be evaluated in this article, or claim that may be made by its manufacturer, is not guaranteed or endorsed by the publisher.

## Supplementary Material

Data Sheet 1 Focus group discussion questionnaire.

Data Sheet 2 Interview guide.
