# Supplementary material for: Factors Associated With Undergraduate Nursing Students' Academic and Clinical Performance: A Mixed-Methods Study
Source: Front Med (Lausanne). 2022 Feb 16;9:793591. doi: 10.3389/fmed.2022.793591 (PMC8889111; doi:10.3389/fmed.2022.793591)
Supplement: Data Sheet 1 — Focus group discussion questionnaire. [file Data_Sheet_1.docx]

Student Questionnaire

Thank you for your participation in this research project on *Factors affecting academic and clinical performance of Bachelor of Nursing students at Monash University*.

Before we commence our group discussion, we would appreciate it if you could take a few moments to fill in this questionnaire.

Thank you for your time. All replies are confidential.

Background INFORMATION

Please place a tick in the appropriate boxes

1. **Age** How old were you when you started your Nursing course?

🞎 <20 yrs old 🞎 20-25 yrs old 🞎 >25 yrs old

1. **Entry Pathway** What education did you complete prior to your Nursing course? Please tick all that apply

| 🞎 Secondary School - Australia | 🞎 Secondary School - International |
| --- | --- |
| 🞎 Technical and Further Education (TAFE) | 🞎 Articulated Program, e.g. Monash College |
| 🞎 Other Entry (Please specify) . . . . . . . . . . . . . . . |  |

1. **Gender** 🞎 Female 🞎 Male 🞎 Other
2. **Campus** 🞎 Clayton 🞎 Peninsula
3. **Enrolment** 🞎 Full-time 🞎 Part-time
4. **Status**
   1. **Domestic Student**: 🞎 HECS 🞎 Full Fee Paying 🞎 Scholarship

- 1. **International Student** 🞎 Full Fee Paying 🞎 Scholarship

1. **What is your country of origin?**
2. **What language/s do you speak at home?**
3. **Do you have family or carer responsibilities at home?**
4. **Are you /have you been in paid employment?** Year 1: 🞎 Full-time 🞎 Part-time

Year 2: 🞎 Full-time 🞎 Part-time

Year 3: 🞎 Full-time 🞎 Part-time

Please turn over the page

student support services

Please place a tick in all the boxes that are relevant to you.

| **During your studies (years 1- 3) have you used any of the following services or supports?** |  | **How often did you use the service or support?**  Please tick the appropriate box for each year level: |
| --- | --- | --- |
| **🞎 Student Academic Support UNit (SASU)** | Year 1  Year 2  Year 3 | 🞎 Never 🞎 Once 🞎 less than 5 times 🞎 more than 5 times  🞎 Never 🞎 Once 🞎 less than 5 times 🞎 more than 5 times  🞎 Never 🞎 Once 🞎 less than 5 times 🞎 more than 5 times |
| **🞎 LIbrary Learning SKILLS Advisors** | Year 1  Year 2  Year 3 | 🞎 Never 🞎 Once 🞎 less than 5 times 🞎 more than 5 times  🞎 Never 🞎 Once 🞎 less than 5 times 🞎 more than 5 times  🞎 Never 🞎 Once 🞎 less than 5 times 🞎 more than 5 times |
| **🞎 HEALTH &Counselling** | Year 1  Year 2  Year 3 | 🞎 Never 🞎 Once 🞎 less than 5 times 🞎 more than 5 times  🞎 Never 🞎 Once 🞎 less than 5 times 🞎 more than 5 times  🞎 Never 🞎 Once 🞎 less than 5 times 🞎 more than 5 times |
| **🞎 English Connect**  **(Let’s Chat)** | Year 1  Year 2  Year 3 | 🞎 Never 🞎 Once 🞎 less than 5 times 🞎 more than 5 times  🞎 Never 🞎 Once 🞎 less than 5 times 🞎 more than 5 times  🞎 Never 🞎 Once 🞎 less than 5 times 🞎 more than 5 times |
| **🞎 English Connect (Assignment Help)** | Year 1  Year 2  Year 3 | 🞎 Never 🞎 Once 🞎 less than 5 times 🞎 more than 5 times  🞎 Never 🞎 Once 🞎 less than 5 times 🞎 more than 5 times  🞎 Never 🞎 Once 🞎 less than 5 times 🞎 more than 5 times |
| **🞎 Peer ASSISTED STUDY SESSIoNS (pASS)** | Year 1  Year 2  Year 3 | 🞎 Never 🞎 Once 🞎 less than 5 times 🞎 more than 5 times  🞎 Never 🞎 Once 🞎 less than 5 times 🞎 more than 5 times  🞎 Never 🞎 Once 🞎 less than 5 times 🞎 more than 5 times |
| **🞎 Informal peer support**  **(E.G. Help from friends/ Support networks)** | Year 1  Year 2  Year 3 | 🞎 Never 🞎 Once 🞎 less than 5 times 🞎 more than 5 times  🞎 Never 🞎 Once 🞎 less than 5 times 🞎 more than 5 times  🞎 Never 🞎 Once 🞎 less than 5 times 🞎 more than 5 times |
| **🞎 individual assistance from Nursing Lecturer/ tutor** | Year 1  Year 2  Year 3 | 🞎 Never 🞎 Once 🞎 less than 5 times 🞎 more than 5 times  🞎 Never 🞎 Once 🞎 less than 5 times 🞎 more than 5 times  🞎 Never 🞎 Once 🞎 less than 5 times 🞎 more than 5 times |
| **🞎 Other - Please specify** |  |  |

**Thank you for your assistance.**
