# Supplementary material for: Factors Associated With Undergraduate Nursing Students' Academic and Clinical Performance: A Mixed-Methods Study
Source: Front Med (Lausanne). 2022 Feb 16;9:793591. doi: 10.3389/fmed.2022.793591 (PMC8889111; doi:10.3389/fmed.2022.793591)
Supplement: Data Sheet 2 — Interview guide. [file Data_Sheet_2.docx]

**INTERVIEW GUIDE: Factors impacted your academic and clinical performance**

**Introduction**

Introduce researcher

Provide participant with a brief explanation of the study.

Ensure that written explanatory information is provided to the participant and the appropriate consent form is completed. Request consent to record the interview

**Body of interview**

*We are interested to understand what factors* have influenced your academic and clinical performance throughout the nursing course.

- What are your perceptions about factors that have influenced your academic performance both in positive and negative ways?
- What are your perceptions about factors that have influenced your clinical performance both in positive and negative ways?
- How receiving learning and social support from university helped you with your academic and clinical performance?
- Could you please share with us some examples?

**Conclude**

Do you have any further questions or queries?

Would you like a summary of this study when it is completed?

May I have your permission to contact you again should I require further clarification of the data?

Thank the participant.
